# Study of Optical Fiber Sensors for Cryogenic Temperature Measurements

**DOI:** 10.3390/s17122773

**Published:** 2017-11-30

**Authors:** Veronica De Miguel-Soto, Daniel Leandro, Aitor Lopez-Aldaba, Juan Jesus Beato-López, José Ignacio Pérez-Landazábal, Jean-Louis Auguste, Raphael Jamier, Philippe Roy, Manuel Lopez-Amo

**Affiliations:** 1 Institute of Smart Cities and Department of Electrical and Electronic Engineering, Campus de Arrosadia S/N, Universidad Pública de Navarra, Pamplona E-31006, Spain; daniel.leandro@unavarra.es (D.L.); aitor.lopez@unavarra.es (A.L.-A.); mla@unavarra.es (M.L.-A.); 2 Department of Physics, Universidad Pública de Navarra, Pamplona 31006, Spain; juanjesus.beato@unavarra.es (J.J.B.-L.); ipzlanda@unavarra.es (J.I.P.-L.); 3 Institute for Advanced Materials (INAMAT), Universidad Pública de Navarra, Pamplona 31006, Spain; 4 Xlim, Fibre Photonics Department, UMR CNRS/University of Limoges 7252, 87060 Limoges Cedex, France; jean-louis.auguste@xlim.fr (J.-L.A.); raphael.jamier@xlim.fr (R.J.); philippe.roy@xlim.fr (P.R.)

**Keywords:** cryogenic temperature, thermometry, interferometric sensor, optical fiber sensor, random distributed feedback fiber lasers, optical backscatter reflectometer

## Abstract

In this work, the performance of five different fiber optic sensors at cryogenic temperatures has been analyzed. A photonic crystal fiber Fabry-Pérot interferometer, two Sagnac interferometers, a commercial fiber Bragg grating (FBG), and a π-phase shifted fiber Bragg grating interrogated in a random distributed feedback fiber laser have been studied. Their sensitivities and resolutions as sensors for cryogenic temperatures have been compared regarding their advantages and disadvantages. Additionally, the results have been compared with the given by a commercial optical backscatter reflectometer that allowed for distributed temperature measurements of a single mode fiber.

## 1. Introduction

Cryogenic temperature systems are becoming more important in the energy sector, transportation, and medicine technology. It is essential to guarantee a safe operation of the systems in these areas providing continuous monitoring of the temperature. However, monitoring at cryogenic temperatures requires greater care than equivalent measurements at room temperature, due to thermal problems of sensor placing and heat sinking.

For the majority of cryogenic applications, resistive, diode, and thermocouple sensors are commonly used [1]. Nevertheless, fiber optic sensors offer many important advantages in contrast to other conventional technologies. On the one hand, some advantages of fiber optic sensors over conventional approaches are their immunity to electromagnetic interferences, compactness, lightweight, relative robustness and durability, and their multiplexing capability, which decreases the total cost of the system [2]. On the other hand, their main drawback is their low intrinsic thermal sensitivity at low temperatures [3], which in fact limits the sensitivity of the sensors at cryogenic temperatures. To overcome this fact, fiber Bragg gratings (FBG) have been widely used [4], but embedded in or bonded to substrates, such as poly methyl methacrylate (PMMA) or Teflon with larger thermal expansion coefficients than silica fibers, which increase their temperature sensitivity [5]. Moreover, metal-coated fiber Bragg Gratings [3,6] and long period gratings inscribed in different type of fibers [7] have been also verified. Although temperature sensitivity is significantly increased, the flexibility of the optical fiber, which is bonded to a rigid substrate, drops using these techniques.

Contrary to FBG sensors, which can be considered as point fiber optic sensors, distributed fiber optic sensing presents unique characteristics over other conventional sensing techniques. The ease to measure temperature and strain continuously along a single fiber is particularly interesting for monitoring large structures, such as pipelines, flow lines, oil wells, and dikes [8], but also for structural health monitoring [9]. A few applications using distributed sensors at cryogenic temperatures have been developed lately, such as distributed liquid level sensors for liquid nitrogen and helium tanks [10], reaching MilliKelvin resolutions in previous works [11].

As far as we are concerned, the use of other types of fiber optic sensors at cryogenic temperatures has not been reported in previous works. In this paper, we report the first demonstrations at cryogenic temperatures of three interferometric and one wavelength selective fiber optic sensor, based on preliminary results presented in [12]. A photonic crystal fiber based Fabry-Pérot sensor, two different Sagnac interferometers, and a random fiber laser sensor that uses a π-phase shifted fiber Bragg grating as sensing element are under study. The behavior of these sensors is compared with a classical FBG sensor and a distributed sensor based on a commercial optical backscatter reflectometer (OBR 4600, LUNA TECHNOLOGIES, Roanoke, VA, USA). The range of the temperature that is analyzed has been extended from 80 °C to −160 °C in order to present a more detailed study on the behavior of these fiber optic sensors, with special emphasis on examining their capabilities and weaknesses. 

## 2. Materials and Methods

### 2.1. Experimental Setup

**Figure 1:**
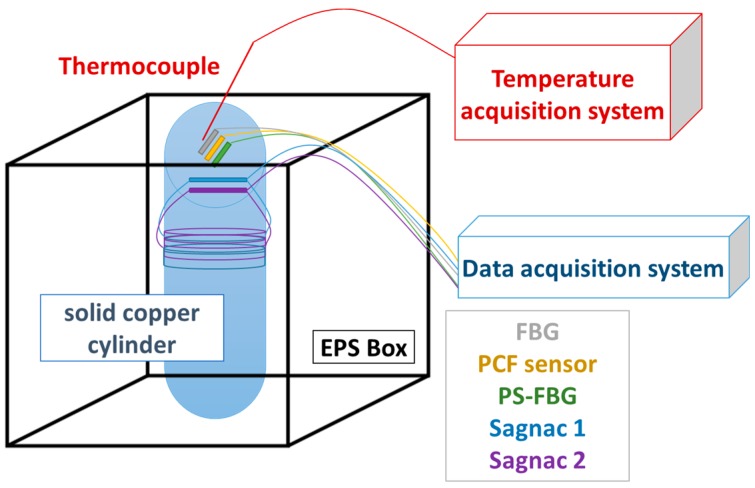
Schematic setup.

The system that is used to attain cryogenic temperatures consists of an Expanded Polystyrene (EPS) box and a solid copper cylinder, which has a high thermal conductivity, which is located inside the box (Figure 1). The diameter and height of the copper cylinder are 4 cm and 5.2 cm, respectively. All of the sensors were analyzed simultaneously in this work. Two wavelength selective fiber optic sensors were employed: A commercial fiber Bragg grating (FBG) and a π-phase shifted fiber Bragg grating (PSFBG) that is interrogated in a random distributed feedback fiber laser (RDFB-FL) configuration. In addition, three interferometric sensors were used: A photonic crystal fiber based Fabry-Pérot (PCF-FP) interferometer and two Sagnac interferometers. Finally, a section of Corning SMF-28 fiber was monitored employing a commercial backscatter reflectometer, obtaining distributed measurements around the copper cylinder. These distributed measurements were performed to compare the obtained results with the given by the other sensors. In addition, knowing the temperature distribution along the copper cylinder may lead to a better understanding of the sensors’ behavior with temperature, as the measuring method is not ideal. 

**Figure 2:**
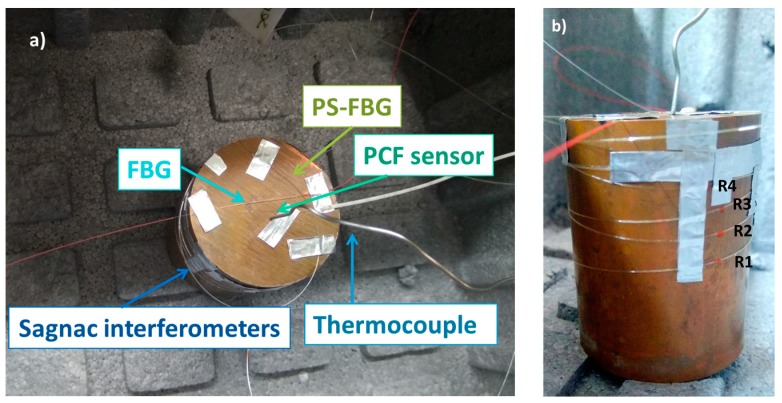
Photographs of the setup: (**a**) placement of the sensors used in the experiment; (**b**) placement of the fiber used for distributed measurements.

In order to have an effective transmission of the temperature, the first three sensors were fixed on top of the cylinder, directly in contact with the copper. Due to their length, the Sagnac interferometers were coiled around the top part of the cylinder, as seen in Figure 2a. A thermocouple type K was placed next to the sensors to provide a temperature reference in real-time. That thermocouple was connected to a data acquisition system where the temperature was recorded every second. The FBG and all of the interferometric sensors were interrogated by a commercial fiber Bragg grating interrogator, Smartec SM125, with a spectral range from 1510 to 1590 nm, a scan frequency of 1 Hz, and a resolution of 5 pm.

In order to study the temperature distribution along the copper cylinder and to have a clear view of the temperature differences inside the setup, distributed measurements have been carried out. Since the transmission of temperature in the copper cylinder is not perfect, several points at different heights have been chosen to verify the temperature variations along the cylinder, taking into account that lower temperatures are expected at the point that is closest to liquid nitrogen. The SMF fiber that was used for distributed sensing was rolled upwardly around the copper cylinder in four loops, as it is shown in Figure 2b. Four measurement points were selected (R1 to R4), forming a vertical line, R1 being at the lowest position (closer to the liquid nitrogen) and R4 at the highest one. 

The aim of the experiment was to prove the feasibility of using different fiber optic sensors to measure cryogenic temperatures, such as a PCF-FP, which has not been previously used for cryogenic measurements, the PSFBG in a RDFB-FL scheme [13], and the Sagnac interferometers [14], which have demonstrated resolutions at room temperature as high as 0.01 °C and 6.2 × 10^−4^ °C, respectively. Although sensitivity is significantly reduced at cryogenic temperatures, because of the low thermal sensitivity of fiber, the high resolution of these sensors could make them suitable to perform measurements at low temperatures. In order to present a more complete comparison of sensors, a commercial FBG has been analyzed, of which the behavior is widely known. With the aim of characterizing the behaviour of the sensors, a temperature sweep has been carried out at higher temperatures. For this purpose, the copper cylinder has been located inside a climatic chamber Binder FD23 together with the sensors, measuring the temperature range between ambient temperature and 80 °C. 

### 2.2. Interferometric Fiber Optic Sensors

The technique that was used to interrogate the interferometric sensors is based on the fast Fourier transform (FFT) of the optical spectrum as in [15]. This interrogation approach has been selected due to the higher resolution offered as compared to conventional techniques (monitoring the wavelength shift of a fringe of the interference) [13]. The FFT of the optical spectra retrieved by the commercial interrogator (SM125, SMARTEC, Lugano, Switzerland) was computed using Matlab every second, presenting real-time information of the sensor system. To monitor the temperature changes, the FFT phase was tracked at the spatial frequency that corresponds to the component generated by each sensing fiber in the FFT amplitude spectrum.

#### 2.2.1. PCF-FP

**Figure 3:**
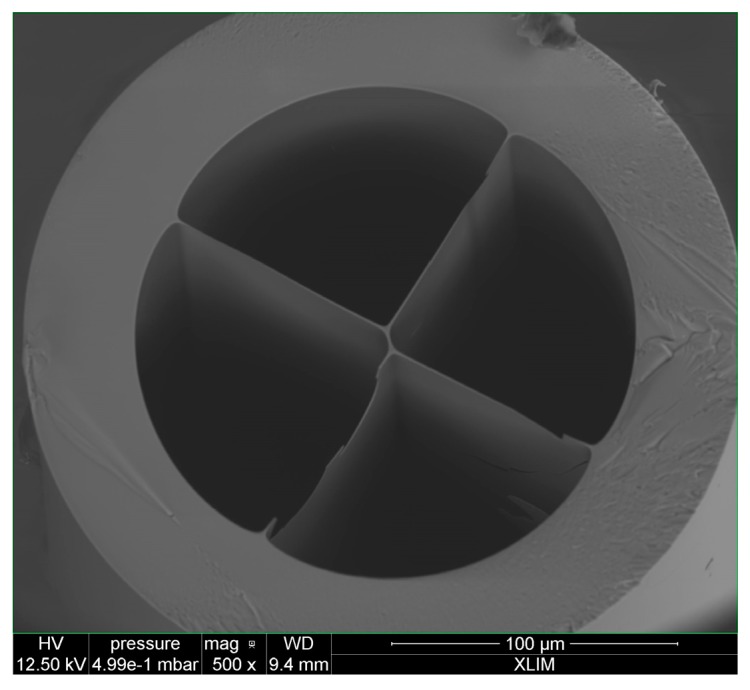
Four-bridge double-Y-shape-core microstructured optical fiber (MOF).

The PCF Fabry-Pérot interferometer that was used in the experiment is formed of a linear cavity made by splicing a standard single mode fiber to a four-bridge double-Y-shape-core microstructured optical fiber (MOF), as shown in Figure 3.

**Figure 4:**
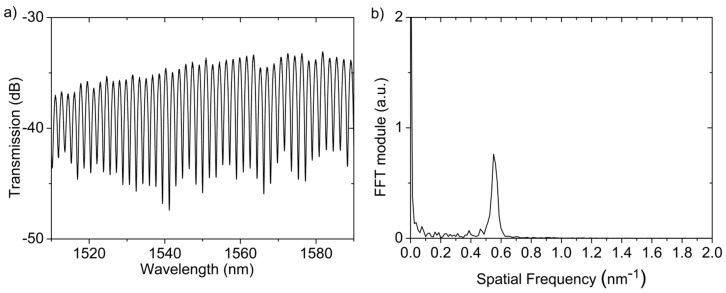
Fiber based Fabry-Pérot (PCF-FP) sensor: (**a**) Optical spectrum and (**b**) its fast Fourier transform (FFT) module.

The MOF was cleaved at the other end, obtaining a cavity length of 570 μm [16]. The optical spectrum of the PCF-FP and its FFT are shown in Figure 4. During the experiment, the FFT phase corresponding to the spatial frequency located at 0.575 nm^−1^ has been monitored. 

#### 2.2.2. Sagnac Interferometers

**Figure 5:**
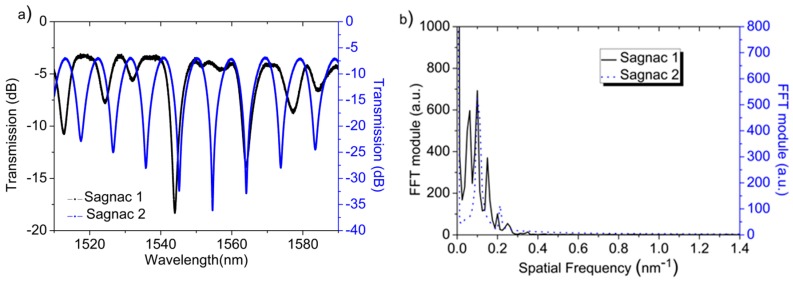
Sagnac interferometers: (**a**) Optical spectrum and (**b**) its FFT module.

Both of the Sagnac interferometers consist of a high-birefringence fiber loop mirror fully made of polarization-maintaining (PM) Panda fiber, which design is based on [15], using the three section analysis. They comprise two PM fibers that act as communication channels fused to the sensing fiber with a 45° rotation angle offset between them. In Sagnac 1, the communication fibers that are used have 0.97 and 0.61m long each, while the sensing fiber is 0.57 m long. The communication fibers that are used in Sagnac 2 have had the same length (0.3 m), while the sensing fiber was 0.45 m long. The optical spectra of the Sagnac interferometers and their FFT are shown in Figure 5. In the first case, it should be noted that, as explained in [14], several components are generated in the FFT magnitude spectrum, corresponding to the sensing fiber, the virtual section given by the different length of the communication fibers and their combination. In the second case, with Sagnac 2, the contributions of the communication fibers are suppressed, being only detected the birefringence changes on the sensing fiber. The temperature measurement is retrieved by monitoring the FFT phase located at 0.125 nm^−1^ and 0.1 nm^−1^ for Sagnac 1 and 2, respectively, which are the contributions that are given by the sensing fibers. 

### 2.3. Wavelength Selective Fiber Optic Sensors

#### 2.3.1. PSFBG in a RDFB-RL Configuration

**Figure 6:**
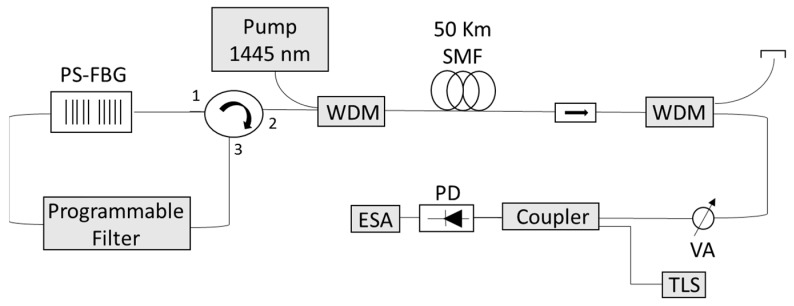
Schematic diagram of the proposed random distributed feedback fiber laser (RDFB-RL) using a π-phase shifted fiber Bragg grating (PSFBG) sensor.

The PSFBG sensor forms part of a forward pumped RDFB-FL scheme, as in [13]. The output of a pump laser (IPG RLD-3-1445,IPG FIBERTECH S.R.L., Legnano, Italy) is injected in the cavity that is formed by 50 km of SMF using a wavelength division multiplexer (WDM) as it can be seen in Figure 6. This fiber forms the active medium for the amplification, due to the stimulated Raman scattering, and also acts as the required distributed mirror for the laser generation through the Rayleigh backscattering effect [17]. The backscattered signal along the fiber is redirected by a 3-port circulator through a programmable filter (Wave Shaper Finisar 1000S), which is centered at the PSFBG wavelength, and through the PSFBG sensor, which acts both as a filter and as a sensor. 

A heterodyne-based detection method was employed to measure the PSFBG wavelength shift since electrical devices offer higher resolution when compared to conventional optical spectrum analyzers. An electrical spectrum analyzer (ESA) tracked the displacement of the beating signal between the output of the laser and an external laser source (TLS) for every degree Celsius.

#### 2.3.2. Commercial FBG

In order to compare the performance of the PSFBG sensor previously explained, the central wavelength of a commercial FBG (1544.9 nm at room temperature) was directly monitored every second employing the FBG interrogator. A commercial FBG with no coating has been chosen to act as a reference, contrary to the one used in [12]. This is because FBGs are representative wavelength selective sensors whose behavior at cryogenic temperatures has been previously studied and is widely known. When comparing its behavior to the ones obtained with the other sensors could give a notion of the reliability of their performance as cryogenic sensors.

### 2.4. Distributed Sensing Using an OBR

**Figure 7:**
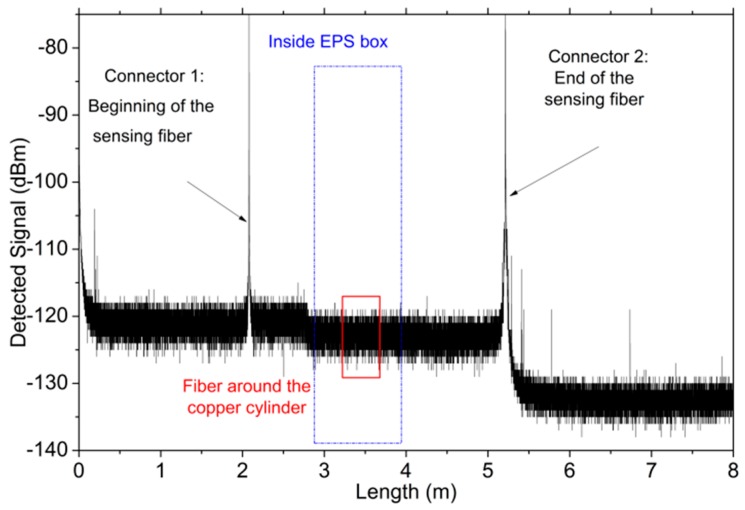
Trace detected by the optical backscatter reflectometer (OBR).

Distributed temperature measurements have been obtained using a three-meter long SMF fiber and a commercial OBR that operates based on optical frequency domain reflectometry [18]. The OBR uses swept wavelength interferometry (SWI) [19] to measure the Rayleigh backscatter as a function of length in optical fiber with ultra-high spatial resolution (10 μm in a span of 30 m) and a backscatter-level sensitivity. If an external stimulus is applied to the sensing fiber (like temperature or strain change), spectral and temporal shifts in the Rayleigh backscatter pattern are induced. When using the OBR for distributed temperature and strain measurements, the device is able to measure these shifts in a span of 70 m, with a spatial resolution of 1 cm, and scales them, giving a distributed temperature or strain measurement, with 0.1 °C and 1 με resolutions, respectively. An example of the reflected signal detected by the OBR that is used in the experiments is shown in Figure 7.

Different sections of the fiber under study can be identified: the first two meters correspond to the pigtail connected to the OBR. Then, a reflection peak at 2 m. determines the position of the first connector and the beginning of the sensing fiber, which ends at 5.2 m. The zone located inside the EPS box is framed in blue (from 2.8 m to 3.9 m), and the region where the fiber is rolled upwards the copper cylinder, which has a total length of around 38 cm, is highlighted in red color.

### 2.5. Cooling Process

Decreasing the temperature of the sensors to cryogenic temperatures was achieved using a simple technique: the EPS box was partially filled up with pure liquid nitrogen, so that the liquid did not reach the sensors. In this manner, the temperature of the copper cylinder gradually decreased. The sensors were placed on the top part of the copper cylinder, which was chosen due to its high thermal conductivity, having a homogenized temperature on its surface. Since the sensors were located close between them, a single thermocouple was used as the temperature reference for all of them.

After adding the liquid nitrogen, the box was covered to avoid the condensation around the sensors, temperature gradients, and also to reduce the evaporation rate of the nitrogen as much as possible, which would lead to fast temperature changes. This fact was verified after several tests. Following this procedure, a stable temperature of −160 °C was achieved. Although liquid nitrogen’s boiling temperature is −195.8 °C at one atmosphere, the copper cylinder could not reach such a temperature because the box was not perfectly isolated, and the transmission of the temperature in the copper cylinder is not ideal. To reach such temperatures, all of the sensors would have need to be submersed in the liquid nitrogen, which in some cases might damage the sensors. 

When the box is filled with liquid nitrogen, the temperature decreases and remains at around −160 °C until all of the nitrogen is evaporated. This process can take several hours. Once the liquid nitrogen is fully evaporated, the temperature rises at around 100 °C/h, until it reaches the room temperature. Measurements have been carried out during the heating process of the copper cylinder, since it is a gradual process without sudden temperature changes. Although this approach is very simple, it cannot reach pure cryogenic temperatures. Several attempts have been carried out to reach lower temperatures using a cryostat and liquid Helium, which presents a boiling temperature of −269 °C. However, the technical difficulties that are associated with the materials used, such as the reduced space and the large temperature gradients in the cryostat, did not allow for obtaining reliable measurements.

## 3. Results

### 3.1. Interferometric and Wavelength Selective Sensors

#### 3.1.1. Characterization at Temperatures below 0 °C

**Figure 8:**
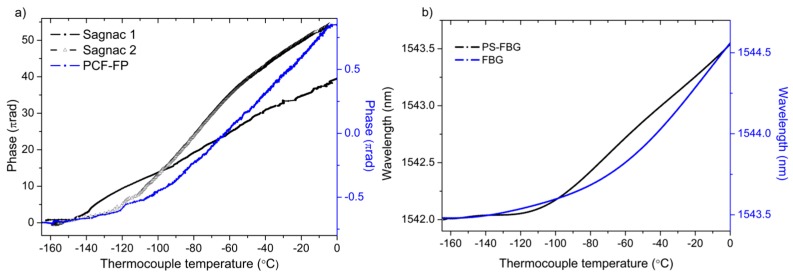
(**a**) Interferometric; (**b**) Wavelength selective fiber optic sensors response versus temperature.

The results of the characterization of the sensors from −160 °C to 0 °C are shown in Figure 8a, for all of the interferometric sensors, and in Figure 8b, for both wavelength selective optical fiber sensors. The measured data is represented as a function of the temperature given by a thermocouple type K, which has a standard limit error of ±2.2 °C.

A summary table gathering some relevant features of the sensors is shown in Table 1, in order to compare their performance as cryogenic sensors. Sensitivity, resolution and response time are analyzed.

It can be inferred from the results shown in Figure 8 that all of the sensors except the Sagnac interferometers present similar behavior with temperature. From 0 °C to −100 °C, a linear trend is observed; nevertheless, from −100 °C to −160 °C, the slopes of the curves are significantly reduced with an exponential decay, becoming almost flat at −160 °C. The fast reduction of the coefficient of thermal expansion below −100 °C [3] justifies this fact, leading to lower sensitivities and resolutions in the measurements. 

Concerning the Sagnac interferometers, two main linear trends are distinguished caused by the slow response of the sensor and the likely coupling of some axial strain, due to the copper cylinder expansion when the temperature increases. In the results shown in Figure 8a regarding both Sagnac interferometers, the effect of the possible parasite strain due to the thermal expansion of the cylinder has not been compensated.

#### 3.1.2. Characterization at Temperatures above 0 °C

**Figure 9:**
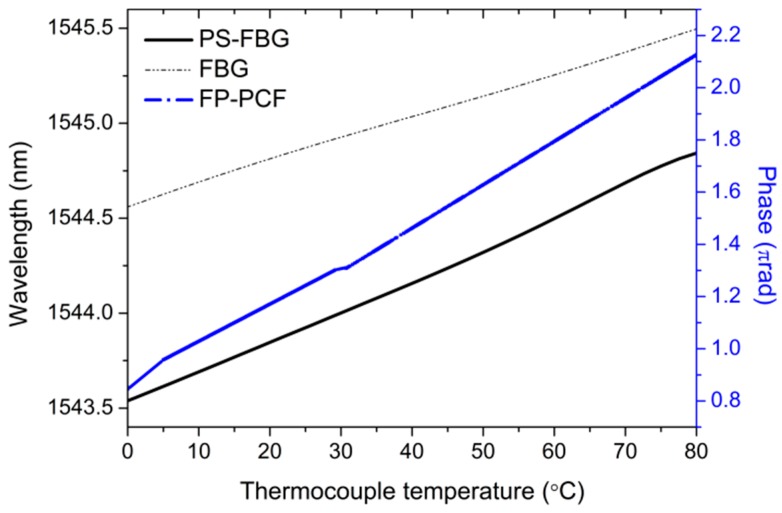
Fiber optic sensors response versus temperature above 0 °C.

The results of the characterization of the sensors from 0 °C to 80 °C are shown in Figure 9 for the FP-PCF and for both wavelength selective optical fiber sensors. The characterization of the Sagnac interferometers have not been included because the measurements with the climatic chamber were not performed under the same conditions with regard to the placement of the sensors that are coiled around the cylinder. The inherent characteristics of the materials that are employed in the experiment did not allow for achieving identical placement conditions. A different location of the sensors would lead to the generation of a parasite strain related with the expansion of the cylinder, altering the measurements.

### 3.2. Distributed Temperature Measurements

**Figure 10:**
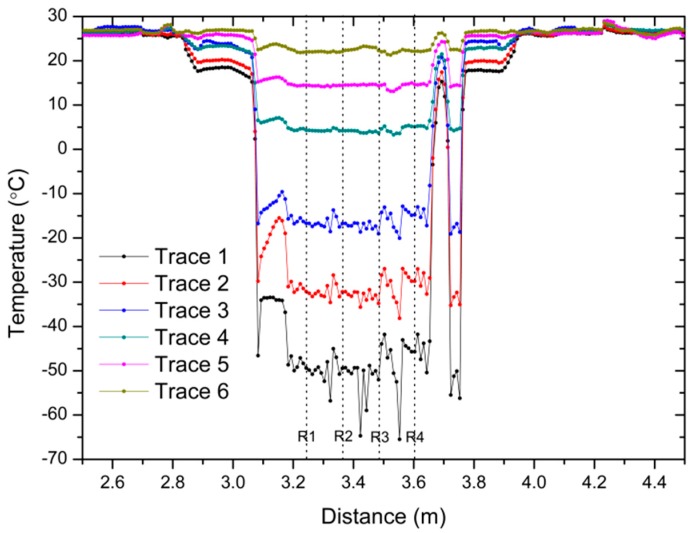
Example of optical backscatter reflectometer (OBR) traces: distributed temperature sensing. R1 to R4 represent the reference points (see also Figure 2b).

**Figure 11:**
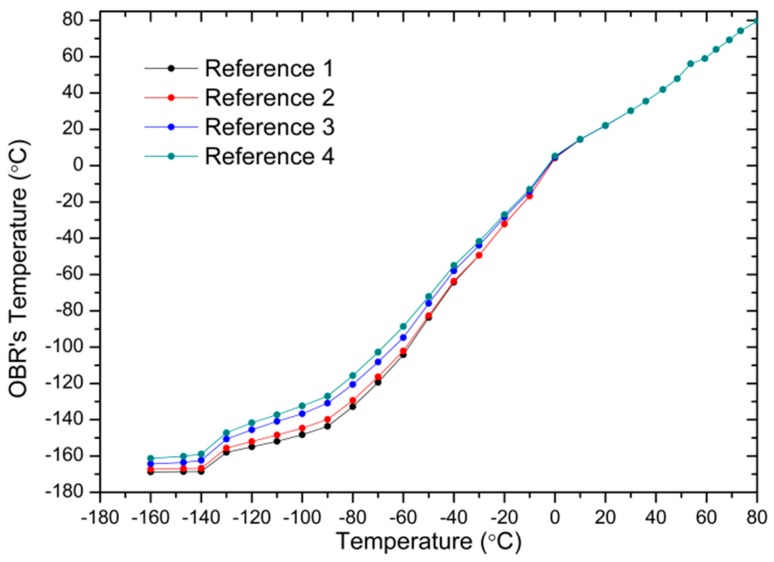
Temperature evolution at the reference points measured with the OBR versus thermocouple temperature.

Distributed measurements have been carried out in a total span of 3 m, with a spatial resolution of 1 cm using a 3 m length SMF fiber and Luna’s Distributed Temperature and Strain Software. The SMF fiber has been rolled upwards the copper cylinder, in order to determine how the temperature varies at different heights of the cylinder. Some examples, represented in different colors, of distributed temperature traces taken during the heating of the cylinder are shown in Figure 10. The temperature evolution of the four reference points along the distributed sensing fiber is represented in Figure 11.

Concerning the measurements showed in Figure 10, despite the good thermal conductivity of the copper cylinder, the zones of the sensing fiber that are closer to the liquid nitrogen are expected to experience lower temperatures, and as the distance from the nitrogen is enlarged, the temperature increases. Different sections can be identified in the graph: the section outside the EPS box is located from 2.5 m to 2.8 m and from 3.9 to 4.5 m. The temperature in this zone remains stable at an ambient temperature (around 25 °C). The area after 2.8 m is located inside the EPS box, but it is not in contact with the copper cylinder, nevertheless it detects the decrease of temperature inside the box, which is almost constant in every point. Finally, the four reference points are located from 3.2 m to 3.6 m, which are in direct contact with the copper cylinder. In this last section, peaks and valleys are observed in specific points, which amplitudes increase at lower temperatures. Since the sensing fiber is attached with aluminum tape to the cylinder, as seen in Figure 2, parasitic strain may be applied to the fiber at these points, originating a noisy profile. However, the shape of the traces remains constant at the four reference points, whose temperature trend has been monitored and represented in Figure 11.

## 4. Discussion

From the multiple experiments that are performed in this work, several key factors are worth mentioning when measuring cryogenic temperatures. Firstly, the response time of the sensors is directly related to the size of the sensor. It stands out the Sagnac interferometers that present a much higher response time, mainly because they are ten times longer than the other sensors (which can be considered as punctual sensors). Another decisive factor is the presence or absence of coating, which also has an impact on the response time of the sensor with temperature. All of the sensors lack of coating except the Sagnac interferometers, presenting as a consequence a higher response time. In addition, it has been noticed a direct influence of the placement of Sagnac interferometers in the setup on the results: the number of turns around the cylinder, the strain caused by the adhesive tape that holds the sensors, the overlap of the fiber turns one on top of another, etc. All of these aspects affect the results, as can be seen in Figure 8a, and need to be considered when using this type of sensors in practical applications at cryogenic temperatures. 

Regarding the sensitivity and resolution, the Sagnac interferometers offer a higher sensitivity and resolution than the PCF-FP because of its higher length. The sensing capability of Sagnac interferometers is based on the phase shift that is generated by the high-birefringence fiber. That phase shift is directly related with the fiber length so longer fiber lengths generate larger phase shifts, i.e. higher sensitivities. As a consequence, employing the same interrogation equipment, higher temperature resolutions can be attained. Superior results were expected when using the Sagnac interferometers, due to the high resolution that was obtained in previous works. However, the use of these sensors at cryogenic temperatures is extremely affected by the placement of the sensing fiber, not being the most suitable sensors for reduced spaces because of the large curvatures to which the fiber is subjected.

On the other hand, the size of the PCF-FP allows for its use in reduced spaces and its placement does not significantly affect the measurements, giving the temperature of a single point at a time. Its characteristics, like the absence of coating and size, give a significantly lower response time to the detriment of the sensitivity and endurance. Condensed vapor inside the sensor may solidify at cryogenic temperatures and cause the breakage of the photonic crystal fiber or modify the interference that occurs in the FP cavity, thus preventing its use.

With regard to the wavelength selective sensors, the PSFBG used in a RDFB-FL configuration and the FBG present analogous sensitivities, which is intrinsically related to the properties of the gratings used and independent from the interrogation technique. Using the heterodyne detection technique of the narrow random laser, two times higher resolution is obtained [9,10]. Below −160 °C, the sensitivity of both the gratings is expected to decrease, and so increase the error in the measurement when using a commercial interrogator. On the contrary, if the heterodyne-based technique in the electrical domain is used, temperatures below −160 °C are expected to be accurately measured. In future works, the results obtained with the PS-FBG could be improved simply by automating the measuring method that is used, since is a limiting factor in dynamic measurements. It should be noticed that enhanced resolution at ultra-low temperatures could be obtained if this measurement technique is combined with coatings and substrates that are applied to the gratings [3,5].

Performing measurements at cryogenic temperatures is complex. Firstly, the reduced spaces needed to perform the measurements might cause large power losses and sometimes damage the sensors due to the difficult handling. The isolation plays an important role since temperature does not remain constant for long periods of time and a fast response of the sensors is required in dynamic measurements. Accordingly, sensors with high response time are not suitable for dynamic measurements at cryogenic temperatures using the aforementioned cooling technique. In addition, temperature gradients in the measuring locations, which are detected by the distributed sensor that is used in the experiment, affect the temperature detected by the sensors, and so the accuracy of the results, which limit the resolution of the measurements.

Regarding the distributed measurements carried out in the experiment, the results confirmed the expected behavior. The temperature of R1, which is located in the lowest position around the cylinder, has experienced the lowest temperature, and as the distance from the nitrogen increases, the temperature also increases. When comparing the OBR’s temperature traces with the temperature that is given by the thermocouple, which is closer to Reference 4, a similar behavior is observed when compared to the sensors analyzed previously. From room temperature to −100 °C all of the curves follow a linear trend. Then, the slope of the curves is reduced and below −140 °C almost any change in temperature is detected. Improved measurements could be obtained by adjusting the scaling coefficients that are used in the Luna’s OBR software for distributed temperature measurements.

## 5. Conclusions

A research on the behavior of five fiber optic sensors at cryogenic temperatures has been performed. In this respect, three interferometric setups (a PCF-FP and two Sagnac interferometers) and two different selective wavelength fiber optic sensors (a commercial FBG and a PSFBG in a RDFB-FL configuration) are studied. As far as the authors know, this is the first time that these interferometric sensors have been proposed to measure cryogenic temperatures. Regarding the all-PM Sagnac interferometers, initial tests show promising capabilities that are mainly due to the high sensitivity and the improved resolution that is given by the FFT interrogation technique. However, there is a trade-off between the length of the sensing fiber, the response time and the size of the sensor. Additionally, further analysis could be done in order to avoid some detected features. On the other hand, the PCF-FP sensor offers limited sensitivity and some fragility, but fast response time and small size.

Promising results have also been given by the PSFBG in the random laser configuration at the cost of the increase in complexity and equipment requirements. Moreover, a study of the temperature profile in the copper cylinder has been performed using a distributed temperature sensor based on a SMF fiber and an OBR, with a spatial resolution of 1 cm in a 70 m range. The results that were obtained are consistent with expectations, following the same trend as the other sensors. Although some differences are observed when compared to the thermocouple temperature, in further work these measurements could be improved by adjusting Luna’s OBR software scale coefficients for distributed temperature measurements.

## Figures and Tables

**Table 1:** Summary of the results.

Type of Sensor	Sensor	Sensitivity above −100 °C	Resolution above −100 °C	Response Time (200 °C Gradient)
Wavelength selective	Commercial FBG	11.6 pm/°C	0.4 °C	~3 s
	PSFBG random laser	9.5 pm/°C	0.21 °C	~3 s
Interferometric	PCF Fabry-Pérot	0.0147 π rad/°C	1.15 °C	~3 s
	Sagnac 1	0.185 π rad/°C	0.38 °C	~15 s
	Sagnac 2	0.4023 π rad/°C	0.1 °C	~15 s
